# Association of croup with asthma in children: A cohort study

**DOI:** 10.1097/MD.0000000000007667

**Published:** 2017-09-01

**Authors:** Sheng-Chieh Lin, Hui-Wen Lin, Bor-Luen Chiang

**Affiliations:** a Department of Pediatrics, Shuang Ho Hospital; b Department of Pediatrics, School of Medicine, College of Medicine, Taipei Medical University; c Graduate Institute of Clinical Medicine, College of Medicine, National Taiwan University; d Department of Mathematics, Soochow University; e Evidence Based Medicine Center, Wan Fang Hospital, Taipei Medical University, Taipei, Taiwan.

**Keywords:** asthma, croup, hospitalization cluster model, risk factors, robust sandwich variance

## Abstract

Asthma and croup are common inflammatory airway diseases involving the bronchus in children. However, no study has reported the effects of urbanization, sex, age, and bronchiolitis on the association of croup and its duration with asthma development. We used the Taiwan Longitudinal Health Insurance Database (LHID) to perform this population-based cohort study; here, the cluster effect caused by hospitalization was considered to evaluate the association between croup and asthma development and the risk factors for asthma in children of different age groups. We evaluated children with croup aged <12 years (n = 1204) and age-matched control patients (n = 140,887) by using Cox proportional hazards regression analysis within a hospitalization cluster. Of all 142,091 patients, 5799 (including 155 with croup [419 per 1000 person-y] and 5644 controls [106 per 1000 person-y]) had asthma during the 5-year follow-up period. During the 5-year follow-up period, the hazard ratios (HRs [95% CIs]) for asthma were 2.10 (1.81–2.44) in all children with croup, 2.13 (1.85–2.46) in those aged 0 to 5 years, and 2.22 (1.87–2.65) in those aged 6 to 12 years. Children with croup aged 7 to 9 years had a higher HR for asthma than did those in other age groups. Boys with croup had a higher HR for asthma. The adjusted HR for asthma was 1.78 times higher in children with croup living in urban areas than in those living in rural areas. In conclusion, our analyses indicated that sex, age, bronchiolitis, and urbanization level are significantly associated with croup and asthma development. According to our cumulative hazard rate curves, younger children with croup should be closely monitored for asthma development for at least 3 years.

## 1 Introduction

Croup, a common respiratory tract disease in young children, involves generalized airway inflammation and edema of the airway mucosa, including that of the larynx, trachea, and bronchus.^[1,2]^ Croup requires clinical diagnosis; it may cause airway obstruction characterized by sudden onset of a distinctive barky cough and accompanied by abnormal breath sounds, hoarseness, and respiratory distress.^[3,4]^ Approximately 3% of all children were estimated to have had croup before the age of 6 years.^[3]^ In the United States, approximately 15% of children visit the emergency department because of respiratory distress.^[5]^ Croup mainly affects children aged <6 years, most frequently occurring at ages of 3 months to 3 years^[1]^; however, it may also occur in adolescents and infants aged <3 months.^[4]^ The symptoms of croup result from airway obstruction mostly caused by acute viral infection. Numerous studies have shown that allergens play a role in recurrent croup.^[1,2,6]^ Children with viral infection develop sensitivity to viral antigens and allergens, leading to recurrent croup.^[5,7]^ Viral infections were detected in approximately 80% of patients with croup.^[5]^ The most common viral infections are parainfluenza 1 and 3 and respiratory syncytial virus (RSV) infection, which exhibit a seasonal pattern.^[3,5,8,9]^

Asthma, also a common respiratory tract disease in children, is characterized by allergy, airway hyperreactivity, and bronchial inflammation.^[10]^ However, viral infection, particularly RSV infection, is also a crucial factor responsible for a considerable proportion of asthma attacks in young children.^[10]^ Smoking and socioeconomic status were reported to be significant risk factors for asthma.^[11]^ Urbanization was also reported to be associated with asthma and acute bronchiolitis in young children.^[10]^ Approximately 37.3% of children diagnosed as having asthma had a family history of croup and asthma.^[12]^ RSV infection was more frequently observed in children with croup and wheezing during the illness than in those without wheezing; children with croup and wheezing during the illness had a significant risk of subsequent and persistent wheezing later in life.^[13]^

No clinical study had reported the risk factors for asthma in children with croup and the effect of the duration of croup on asthma development. We hypothesized that urbanization, sex, age, and bronchiolitis were independent risk factors for asthma in children with croup. In this population-based study, we considered the hospitalization effect to evaluate the relationship between croup and asthma development.

## 2 Patients and methods

### 2.1 Study population and design

The National Health Insurance (NHI) program of Taiwan is a mandatory social insurance system. This single-payer program was launched on March 1, 1995, covering most of the residents Taiwan. By 2007, approximately 22.60 million of Taiwan's 22.96 million residents enrolled in this program. Foreigners living in Taiwan are also eligible for the program. The program provides health care services for illness, injury, and maternity. The Longitudinal Health Insurance Database (LHID) contains registration files and original reimbursement claims data for 1 million selected beneficiaries of the NHI program. The Taiwanese Bureau of National Health Insurance Administration collects these data, including International Classification of Diseases, Ninth Revision, Clinical Modification (ICD-9-CM) codes, from the NHI program and sorts them into files; these are provided to scientists for research purposes.^[14]^ Because the LHID constitutes de-identified secondary dataset that were analyzed anonymously, the need for informed consent of the study was waived.

Data regarding the study population were extracted from the Taiwan LHID2005, which contains all original claims data of 1 million patients who were randomly sampled from the 25.56 million insurants in the 2005 registry of the NHI Research Database; thus, the LHID2005 is a large and reliable database, and numerous studies using LHID2005 data have been published in peer-reviewed journals.^[14]^

Our croup cohort comprised all patients with croup (ICD-9-CM 464.4). Because croup mainly affects children aged <6 years,^[1]^ we included children aged 6 to 12 years in the comparison group. We stratified children aged <12 years into different age groups and determined the relationship between croup and asthma development at a usual age (0–5 y) and an unusual age (6–12 y). All patients aged >12 years were excluded. In total, the cohort consisted of 1204 patients with croup divided into 2 subgroups: usual age (0–5 y) and unusual age (6–12 y). The remaining 140,887 patients in the LHID2005 aged ≤12 years were included in the control cohort. The 359 cities or towns in Taiwan were stratified into 7 levels according to National Health Research Institutes standards to classify patients according to the urbanization level—from Level 1 (most urbanized) to Level 7 (least urbanized). In this study, we combined Levels 1 and 2 as the urban group, Levels 3 and 4 as the suburban group, and Levels 5, 6, and 7 as the rural group.^[10]^

### 2.2 Outcome variable

We selected asthma (ICD-9-CM 493) as the outcome variable, and all *control* cohort patients were diagnosed as having asthma during the follow-up period. In Taiwan, a diagnosis of asthma is based on the Global Initiative for Asthma guidelines 2004.^[15]^ According to the regulations of the National Health Insurance Institutes in Taiwan, children diagnosed as having asthma should have positive data describing their medication and family histories, atopy histories, total immunoglobulin E (IgE) counts, allergen-specific and lung function test results, and asthma control scores. Patients in the croup and control cohorts were individually followed up for asthma diagnosis from January 1, 2004, to December 31, 2008.

### 2.3 Statistical analysis

**Figure 1:**
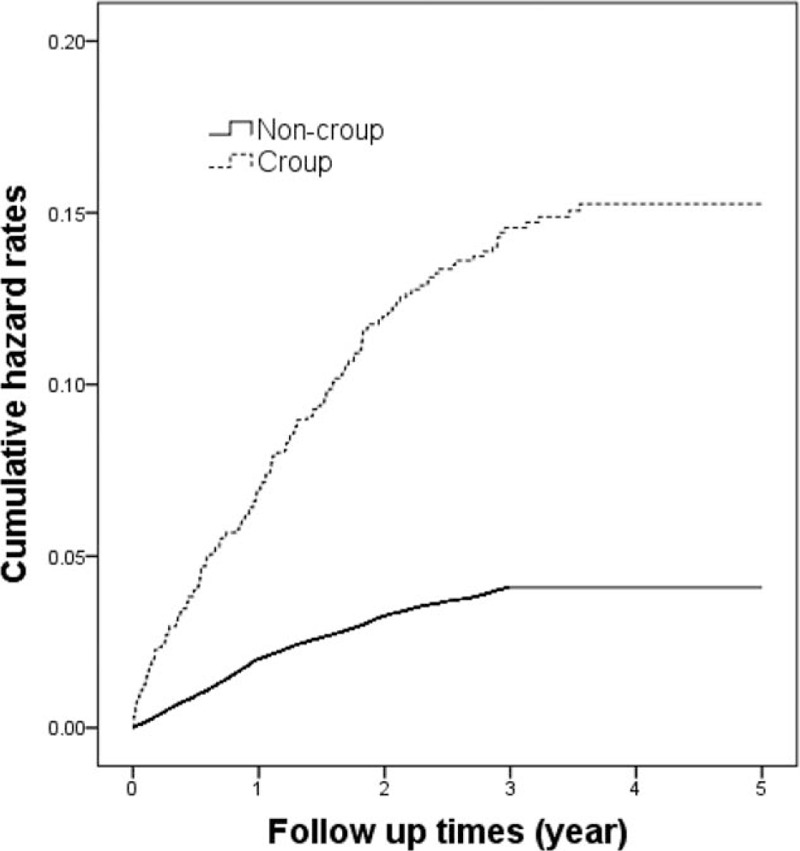
Cumulated hazard rate curves based on Kaplan–Meier analysis.

The effects of different hospitals on the outcomes of this study were considered. The asthma-free survival rates were evaluated using a Cox model; we also used log–log plots to confirm compliance with Cox model assumptions. Hazard ratios (HRs) for asthma were compared between the cohorts after adjustment for patient age (continuous), sex, bronchiolitis status, and urbanization level (Table 1). In addition, we stratified the analysis to clarify the association between croup and asthma development (Table 2), because the model for the age–croup relationship (Table 1) showed a significant correlation. The robust sandwich covariance matrix estimation method, detailed by Lin and Lin^[16]^ in their appendix, was employed. Pearson's chi-squared test and Student's *t* test were used to compare group differences among categorical and continuous variables, respectively (Table 3). The Kaplan–Meier method was used to estimate the cumulative hazard rate curves, and the log-rank test was used to examine the differences in the risk of asthma between the cohorts (Fig. 1). Data analyses were conducted using the SAS statistical package (version 9.1.3; SAS Institute Inc., Cary, NC), and a *P* value of <.05 was considered significant.

**Table 1:**
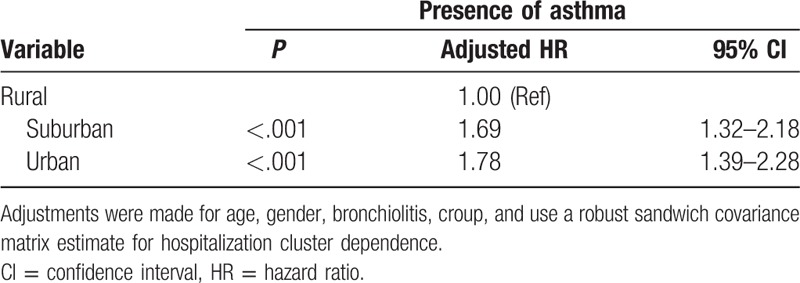
Adjusted hazard ratios (HRs) and 95% confidence intervals (CIs) for asthma among the urbanization during the up to 5-year follow-up period (N = 142,091).

**Table 2:**
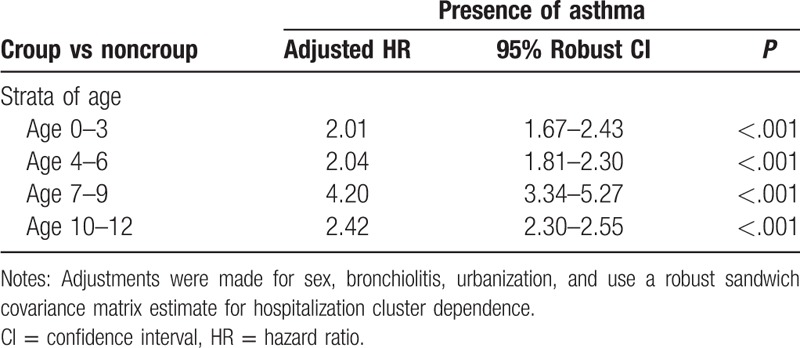
Adjusted hazard ratios (HRs) and 95% confidence intervals (CIs) for asthma among the croup vs noncroup children in different strata of age (N = 142,091).

**Table 3:**
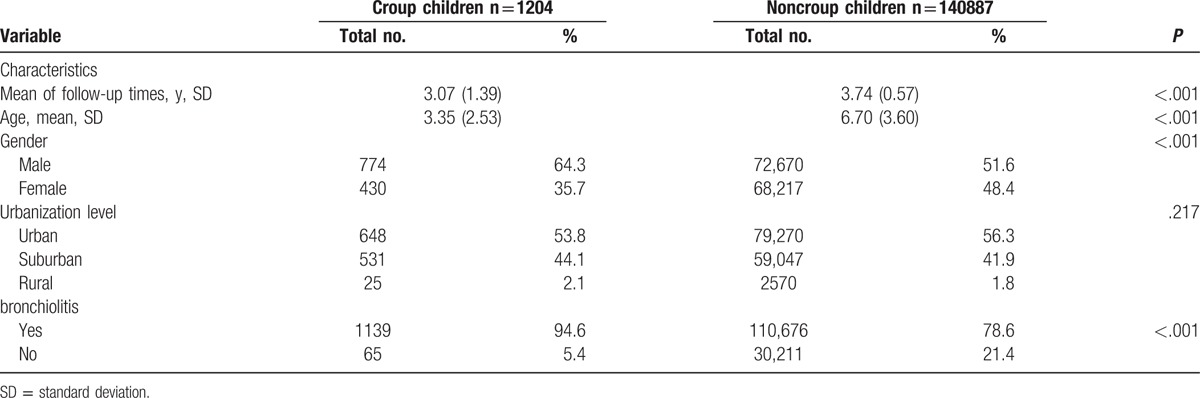
Demographic characteristics for croup and noncroup children in the cohort, 2004–2008, age 0–12 (N = 142,091).

## 3 Results

In total, 1204 children with croup and 140,887 controls were enrolled in this study. The baseline variables for the urbanization level showed no significant difference between the cohorts (Table 3). As shown in Fig. 1, the hazard rate curves, plotted using the Kaplan–Meier method, indicated a higher risk of asthma in the croup cohort than in the comparison cohort during the follow-up period (*P* < .001).

Data regarding asthma diagnosis among the croup and comparison cohorts are presented in Table 4. Of all 142,091 patients, 5799 (including 155 with croup [419 per 1000 person-y] and 5644 controls [106 per 1000 person-y]) had asthma during the 5-year follow-up period. As shown in Table 3, a significant difference was observed in the incidence of asthma according to age in the cohorts (*P* < .001). To evaluate the deviation caused by age, we subdivided the patients into 2 age groups. We then estimated the asthma development rates in children with croup to be 470 per 1000 person-years for those aged 0 to 5 years and 200 per 1000 person-years for those aged 6 to 12 years during the 5-year follow-up period. In the Cox proportional hazards regression analysis, the adjusted HRs (95% CIs) for asthma during the 5-year follow-up were 2.10 (1.81–2.44) in all patients with croup, 2.13 (1.85–2.46) in those aged 0 to 5 years, and 2.22 (1.87–2.65) in those aged 6 to 12 years, compared with age-matched comparison cohort patients. Table 1 lists the adjusted HRs for asthma based on the multivariable Cox proportional hazards regression analysis. According to urbanization levels, the adjusted HRs (95% CIs) for asthma during the 5-year follow-up were 1.69 (1.32–2.18) in the suburban group and 1.78 (1.39–2.28) in the urban group, compared with the rural group. Table 2 lists the adjusted HRs for asthma among the different age groups. We stratified the groups by age to further clarify the association between croup and asthma development. In addition, the cluster effect caused by hospitalization was considered, and a robust estimation method was used to obtain the HRs and 95% CIs. The adjusted HRs for asthma were significantly higher in children with croup than in those without in all age groups, particularly in those aged 7 to 9 years (HR 4.20, 95% CI 3.34–5.27).

**Table 4:**
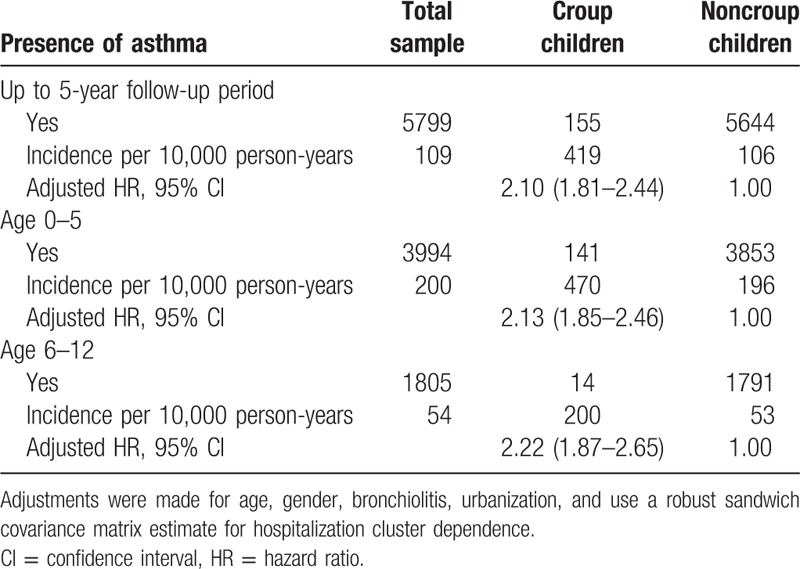
Incidence and 95% confidence intervals (CIs) for asthma among the croup children and noncroup children during the up to 5-year follow-up period (N = 142,091).

## 4 Discussion

Asthma is a common obstructive respiratory disease in children, characterized by decreased forced expiratory volume in 1 second predicted through spirometry. The etiology of asthma is complex, involving allergy, immunological responses, genetics, and environmental exposure.^[10]^ Viral infection, a higher body mass index, parental asthma, smoking, socioeconomic status, allergens, pets, prematurity, and current environment have been reported as potential risk factors for asthma.^[10,11,16,17]^ Moreover, viral infection, eczema, and a family history of hay fever and chronic bronchitis have been revealed to be associated with croup.^[5,7,18]^ Children with croup had a lower mean percentage predicted forced expiratory flow at both 50% and 75% of forced vital capacity than did those without croup.^[18]^ In this study, we assumed that croup is associated with asthma development and investigated several variables to establish whether they are risk factors for asthma in children with croup. We analyzed patients of different ages, sexes, bronchiolitis statuses, and urbanization levels. Cherry^[1]^ reported that the incidence of croup in boys is approximately 1.5 times higher than that in girls. We observed that croup was more common among boys compared with girls in Taiwan at a ratio of 1.8. Boys with croup were more susceptible to asthma than were girls (HR 1.37). A study revealed that neither croup nor bronchiolitis was related to asthma development, but it included only 91 children.^[19]^ In children with croup having initial lower airway involvement, abnormal lung function may increase the risk of any type of recurrent wheezing in the lower respiratory tract.^[13]^ Castro-Rodríguez et al^[13]^ reported that croup with wheezing significantly increased with lower airway obstruction, with odds ratios of 4.2, 3.4, and 3.2 at the ages of 6, 8, and 11 years, respecitvely.^[13]^ In our study, during the 5-year follow-up period, the HR for asthma was 2.10 in the croup cohort compared with the control cohort. In this study, because we used a large sample size, the association between croup and asthma development was confirmed. In the usual age (0–5 y) group, the adjusted HR for asthma was 2.13 compared with the control cohort; in the unusual age (6–12 y) group, the adjusted HR for asthma was 2.22 compared with the control cohort. Thus, children developing croup at an unusual age had a higher adjusted HR for asthma than did those developing croup at a usual age; however, children developing croup at a usual age had a higher incidence of asthma than did those developing croup at an unusual age (470 vs 200 per 10 000 person-y). We divided the children into subgroups to identify the age group at the highest risk of asthma among patients with croup. As shown in Table 2, the adjusted HRs for asthma in children with croup aged 0 to 3, 4 to 6, 7 to 9, and 10 to 12 years were 2.01, 2.04, 4.20, and 2.42, respectively. Therefore, we suggest that children with croup at an unusual age of 7 to 9 years should be carefully monitored for signs of asthma. Bronchiolitis was associated with asthma in young children in a previous study.^[10]^ In the current study, bronchiolitis was noted after the first episode of croup; bronchiolitis was also significantly associated with croup and asthma. In our analyses of the effects of urbanization levels, the prevalence of croup was similar among children living in urban areas (prevalence 0.008) and rural areas (prevalence 0.009). However, the HRs for asthma in children with croup living in urban and suburban areas were 1.78 and 1.69, respectively, compared with those in rural areas. We noted that children with croup and asthma living in urban areas had a higher HR than did those in rural areas. We inferred that children living in urban areas may have more allergies and more hyperreactive airways because of higher air pollutant and environmental allergen exposure. Therefore, when children living in urban areas developed croup due to viral infection, their airways may have had increased allergic, inflammatory, and immune responses, leading to asthma development. A previous study^[20]^ revealed that seasonal variation is a factor influencing hospitalization rates for croup; considerably high hospitalization rates were observed among boys aged <1 year. Of children who visited the emergency department with croup symptoms, approximately 85% had mild croup and only 1% to 8% required hospitalization.^[5,21]^ Although croup is mild and self-limiting, some patients occasionally experience severe airway obstruction, with 1.3% to 2.6% of them requiring hospitalization.^[2]^ Fewer than 3% of children with croup were hospitalized and required intubation.^[5,21]^ Hospitalization may cause a cluster effect influencing the relationship between croup and asthma. To minimize this effect, we used a robust sandwich covariance matrix estimation method for hospitalization cluster dependence. According to the asthma hazard rate curves obtained in our study, we suggest that children with croup should be followed regularly and observed closely for at least 3 years. Parents should be educated to prevent recurrent viral infections and exposure to allergens and advised to monitor their children for possible symptoms of asthma.

The physiological association between croup and asthma remains unclear. The standard treatment for asthma involves corticosteroids.^[15]^ Croup management involves the maintenance of a reasonable fluid intake; use of antipyretics, aerosolized epinephrine, and steroids; and endotracheal intubation for children progressing to respiratory failure.^[3,22]^ Corticosteroid use in children is the oldest and most effective treatment of croup.^[4,12]^ A study in 2006 focused on treatment with oral, intravenous, or nebulized corticosteroids.^[3]^ Several trials of corticosteroids involving various drugs, dosages, and routes of administration for croup treatment have been conducted.^[1]^ Oral steroids and nebulized steroids, such as budesonide, are effective in treating children with croup in outpatient and inpatient settings and are a safe alternative treatment of moderate-to-severe croup.^[6,23–24]^ Low doses of oral dexamethasone (0.15 and 0.30 mg/kg) have been reported to have the same effect as the standard dose of 0.6 mg/kg on children with croup.^[3,25]^ Both asthma and croup symptoms worsen at night; physiologically plausible explanations are the occurrence of gastroesophageal reflux at night and circadian fluctuations, which reduce endogenous serum cortisol levels between 23:00 and 04:00 observed in children.^[4,26–28]^ A study^[5]^ revealed that treating gastroesophageal reflux improved the respiratory symptoms of recurrent croup. Therefore, gastroesophageal reflux and serum cortisol levels possibly have the same physiological effects in croup and asthma.^[28]^ Recent studies on croup or asthma have focused on genetic factors. Because *CD14* plays a crucial role in signaling the innate immune response and detecting inflammation-provoking pathogens, Rennie et al^[29]^ studied the 2 polymorphisms of *CD14* through haplotype analyses and reported that people with *CD14* variants of the TT haplotype were significantly more prone to croup and asthma. Therefore, genetic factors may influence croup and asthma.

The study has a possible limitation. The diagnoses of croup and asthma were completely determined using the ICD-9-CM codes listed in the NHI Claims database; however, concerns may be raised regarding the diagnostic accuracy of the database. Nevertheless, the sample size was sufficiently large to mitigate the possible bias.

## 5 Conclusion

Children with a history of croup have a high risk of asthma. Furthermore, children with croup living in urban areas, of the male sex, or aged 7 to 9 years have an increased risk of asthma. Parents must be educated that all children with croup should be monitored for asthma development for at least the first 3 years.
